# Eutectic Mixtures Based on Oleic Acid and Pulsed Electric Fields: A Strategy for the Extraction of Astaxanthin from Dry Biomass of *Xanthophyllomyces dendrorhous*

**DOI:** 10.3390/foods14132371

**Published:** 2025-07-04

**Authors:** Javier Marañés, Alejandro Berzosa, Fernando Bergua, Javier Marín-Sánchez, Javier Raso, Manuela Artal

**Affiliations:** 1 Departamento de Química Física, Facultad de Ciencias, Instituto Agroalimentario de Aragón-IA2, (Universidad de Zaragoza-CITA), 50006 Zaragoza, Spain; javiermaranes@gmail.com (J.M.); ferber@unizar.es (F.B.); martal@unizar.es (M.A.); 2 Food Technology, Facultad de Veterinaria, Instituto Agroalimentario de Aragón-IA2, (Universidad de Zaragoza-CITA), 50013 Zaragoza, Spain; aberzosa@unizar.es (A.B.); j.marin@unizar.es (J.M.-S.)

**Keywords:** astaxanthin, pulsed electric fields, hydrophobic eutectic solvents, extraction

## Abstract

The use of astaxanthin (AST) is expanding from its origins as a food coloring to health-related applications. This paper evaluates the efficiency of its extraction from dried *Xanthophyllomyces dendrorhous* using two combined and consecutive techniques. First, cell membrane permeation is achieved with the application of pulsed electric fields (PEFs). Solid–liquid extraction is then performed with hydrophobic eutectic solvents (hESs) prepared by mixing components of essential oils (linalool, l-menthol, eugenol, geraniol, cinnamyl alcohol, or thymol) and oleic acid. The hESs were characterized by measuring of several thermophysical properties at 25 °C and 0.1 MPa. An initial screening was performed to choose the best solvent and the extraction conditions (composition, extraction time, and temperature) were evaluated using the response surface methodology. The results showed the importance of the electroporation as a preliminary step to the extraction. The more hydrophobic and less compact the hES, the more effective the solvent. Thus, the equimolar mixture of l-menthol and oleic acid achieved an efficiency of 77% for untreated biomass, 83% for that treated with PEF, and 92% for that treated with PEF and later incubated. Molecular dynamics simulations demonstrated the importance of the hydrophobic interactions between AST and the components of the best solvent.

## 1. Introduction

Astaxanthin (AST) is currently one of the most promising carotenoids due to its interesting physicochemical properties and positive physiological effects on health. From a commercial perspective, the compound annual growth rate of AST is up to 16% of the expanding market value. Regarding scientific interest, the number of published papers with the word “astaxanthin” as the topic (Web of Science) was 95 in 2000 and 759 in 2024. This information and a comprehensive review on the characteristics and uses of the AST has been published by Nishida et al. [1]. Briefly, the AST (3,3′-dihydroxy-β,β-carotene-4,4′-dione) is a carotenoid whose molecular structure includes two hydroxyl and two carbonyl groups. It has three stereoisomers owing to the presence of two chiral carbons and 512 geometric isomers due to the nine double bonds in the polyene moiety. The structure also includes 13 conjugated double bonds that give it an intense red-orange color. For the latter, the classic use of this compound has been as a colorant in fisheries, poultry, and livestock sectors. The conjugated π-electron chain is also responsible for the high antioxidant power of AST. Other health benefits related to cardiovascular, neurological, and metabolic diseases have been reported in the literature.

The efficient obtaining of AST of natural origin is a procedure with several stages. The first is the culture of the matrix. The most common natural sources of AST are the microalgae *Haematococus pluvialis* and yeast *Xanthophyllomyces dendrorhous* (asexual reproductive stage of *Phaffia rhodozyma*). The alga has higher AST performance and produces the (3S,3′S) enantiomer mainly in ester form. The yeast is easier to grow and free (3R,3′R) enantiomer is obtained from it. The properties of the optical isomers are different; therefore, this may sometimes be an aspect to consider when choosing the source. After, a pretreatment of the biomass that facilitates the release of intracellular compounds is convenient. Novel techniques using high pressure, microwaves, ultrasound, or electric fields are being evaluated to disrupt the lipophilic cell wall avoiding the traditional use of solvents such as the dimethyl sulfoxide, acetone, or hexane [2,3,4,5,6]. Once the biomass is prepared, a solid–liquid extraction is performed for which the choice of the appropriate solvent is an essential step. Among other properties, a low impact on the environment must be required so neoteric solvents such as supercritical fluids, bio-based, or eutectic solvents are proposed [7,8]. It is currently considered that a sustainable process must also include the recycling stage of the solvent used. Different procedures are studied for the reuse of eutectic solvents such as the use of antisolvents, switchable solvents, and evaporation, among others [9,10]. However, all of them involve an energy cost and/or an increase in waste, which contributes negatively contributes to the environmental assessment of the global process.

An alternative proposal is to use natural hES whose composition results in a ready-to-use extract with improved properties [8,11,12,13,14]. Depending on the components, the antioxidant capacity, solubility, dermal penetrability, and stability of the extract can be increased [10,15,16]. This is a promising avenue that should be explored as it reduces the costs and hassle of separation and recycling processes. Along these lines, solvents composed of oleic acid (Oa) and components of the essential oils (EOs) can provide the final formulation containing AST, a series of characteristics that are especially interesting for the biotechnological industry. All of them are bioactive compounds with important biological properties such as anti-inflammatory and anticancer activity, mild analgesics, and powerful antimicrobials and antioxidants. Therefore, its use in the prevention of diseases and as cosmeceutical and nutricosmetic substances is growing.

Essential oils have interesting functional properties but their direct incorporation into foods is unfeasible due to their high organoleptic effects. Nevertheless, synergic effects can be found by mixing small amounts of EOs with other natural functional compounds notably improving the film properties [17,18]. For instance, Oa is an efficient natural plasticizer and a good antioxidant and antimicrobial [19]. Carotenoids are also interesting additives [20]. Results of the combination of all of them in the formation of biofilms have already been reported in the literature. Mussagy et al. [13] extracted AST from yeast with a hydrophilic eutectic composed of choline chloride and butyric acid as an extracting solvent. They demonstrated that this acidic eutectic mixture containing carotenoids is ready for use in the manufacturing of bioactive biodegradable films. The film showed high efficiency in preventing the shelf life of strawberries [21].

In this context, the extraction of astaxanthin from freeze-dried *X. dendrorhous* yeast by combining the PEF technique as a cell disruption method and the use of hydrophobic eutectic mixtures as ecological solvents was evaluated. For that, six binary mixtures of oleic acid and components of essential oil were prepared and their main thermophysical properties were determined. The microorganism was cultivated and the suspension was treated. The biomasses obtained were characterized by measuring the content of different compounds and their antioxidant capacity. Later, a screening of these mixtures was performed to select the most effective solvent. Once performed, an optimization of the extraction conditions was carried out by the response surface methodology (RSM) approach. Finally, the interactions between astaxanthin and the most effective solvent were analyzed using molecular dynamics simulations.

## 2. Materials and Methods

### 2.1. Biomass Cultivation and Treatment

#### 2.1.1. Strain, Media, and Growth Conditions

The *Xanthophyllomyces dendrorhous* strain ATCC^®^ 74219™ was sourced from the American Type Culture Collection (ATCC, Beltsville, MD, USA). Following recovery, the strain was preserved in 20% glycerol at −80 °C for long-term storage, and on Potato Dextrose Agar (PDA, Oxoid, Basingstoke, UK) plates at 4 °C for short-term storage. A pre-culture was prepared by isolating a single colony from the PDA plates and growing it in 25 mL of Potato Dextrose Broth (PDB, Oxoid, Basingstoke, UK) in a 100 mL Erlenmeyer flask on a rotary shaker (Heidolph Unimax 1010, Schwabach, Germany) at 270 rpm and 25 °C for 2 days. AST was produced by inoculating 250 mL of PDB in a 500 mL Erlenmeyer flask with a cell density of approximately 10^6^ cells/mL. *X. dendrorhous* cells were then incubated for 6 days at 25 °C with continuous agitation (270 rpm) [22].

#### 2.1.2. PEF Treatment

Prior to PEF treatment, the cell biomass obtained from the culture was centrifuged at 6000× *g* for 5 min at 20 °C (Heraeus Megafuge 1.0R, Newbury, UK) and resuspended in a citrate-phosphate McIlvaine buffer (pH 7, conductivity 2 mS/cm) to reach a final concentration of 3 × 10^−3^ g dry weight/mL.

Cells were electroporated using a commercial PEF generator (Vitave, Prague, Czech Republic). Monopolar square wave pulses of 3 µs width were applied in a parallel titanium electrode chamber (0.4 cm electrode distance, 3.0 cm length, and 0.5 cm width). The cell suspension was pumped through the chamber at a flow rate of 5 L/h using a peristaltic pump (BVP, Ismatec, Wertheim, Germany). To achieve electroporation of over 90% of *X. dendrorhous* cells, 45 pulses at 20 kV/cm (54 kJ/kg) were applied [22].

Following PEF treatment, the suspension was divided into two aliquots. One aliquot was immediately centrifuged at 6000× *g* for 5 min at 4 °C and the pellet was frozen at −40 °C. The second aliquot was incubated in the same treatment buffer in darkness for 24 h at 25 °C prior to centrifugation and freezing under the same conditions.

**Figure 1:**
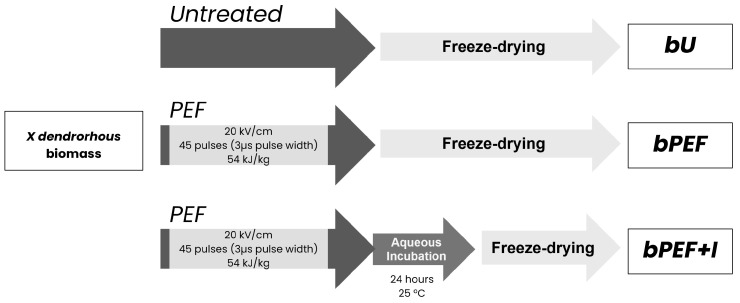
Experimental workflow for sample preparation. *X. dendrorhous* biomass was either left untreated (*bU*), subjected to PEF treatment (*bPEF*), or subjected to PEF followed by a 24 h incubation at 25 °C in buffer (*bPEF + I*). All samples were subsequently freeze-dried before extraction.

To evaluate the effect of PEF treatment and post-treatment incubation on the extraction of AST using eutectic mixtures, three sample types were prepared: untreated biomass (*bU*), biomass immediately after PEF treatment (*bPEF*), and biomass obtained after PEF treatment followed by 24 h incubation in darkness at 25 °C (*bPEF + I*) (Figure 1). Resulting freezing pellets were freeze-dried (FreeZone 6, Labconco, Kansas City, MO, USA), and ground into fine powder using a mortar and pestle prior to further analysis.

### 2.2. Biomass Characterization

#### 2.2.1. Determination of Dry Weight

One milliliter of each aliquot (*bU*, *bPEF*, *bPEF + I*) in a conical flask was washed three times with distilled water, with each wash involving centrifugation at 6000× *g* for 10 min at 20 °C. After washing, the distilled water was removed, and the pellet was dried in a centrifugal concentrator (miVac DNA-23050-B00, Ipswich, England) at 4000× *g* and 30 °C until a constant weight was achieved (approximately 20 min). The difference in weight between the conical flasks containing the wet and dry biomass was used to calculate the dry cell weight, expressed as g_wet_/g_dry_.

#### 2.2.2. Determination of Glutathione (GSH) Content

The colorimetric method with 5,5′-dithiobis-2-nitrobenzoic acid (DTNB) was used to determine the content of reduced form of GSH in the supernatant of the *X. dendrorhous* culture. It is based in the reaction of GSH and DTNB to produce the yellow chromophore 5′-thio-2-nitrobenzoic acid (TNB) measurable at 412 nm. The procedure was similar to that described by Ganeva et al. [23]. Two solutions were prepared with phosphate buffer solution (PBS) of pH = 7.5 as solvent: ethylenediaminetetraacetic acid (EDTA) 5.6 mM and DTNB 0.4%. The purities of the pure EDTA and DTNB were ≥99% and >98%, respectively. After, 960 μL of EDTA solution, 20 μL of DTNB solution, and 20 μL of supernatant sample were mixed and incubated for 10 min at room temperature. Finally, the absorbance of these mixtures at 412 nm was determined. The values were compared with the calibration curve (see Supplementary Materials) made with GSH (≥98%) in ethanol in a concentration range of 0.004 to 2.000 mg/mL. The results are expressed as mg_GSH_/g_b_.

#### 2.2.3. Determination of Free α-Amino Nitrogen (FAN) Content

The ninhydrin assay described by Dimopoulos et al. [24] was used to evaluate the FAN concentration in biomass. Ninhydrin induces the oxidative decarboxylation of amino acids that have the free amino group. The reaction products are ammonia, carbon dioxide, and hydrindantin. The latter in turn reacts with the produced ammonia and another molecule of ninhydrin given a complex compound. This blue-purple complex strongly absorbs at 570 nm. For each biomass type, a volume of 500 μL of supernatant was mixed with 250 μL de ninhydrin reagent and incubated at 100 °C for 15 min. After, the suspension was cooled in an ice bath for 5 min and the reaction was stopped by adding a solution of 0.2% of KIO_3_ in diluted ethanol (40%). The absorbance at 570 nm was measured with distilled water as blank. The values were compared with the calibration curve (see Supplementary Materials) made with L-alanine (≥98%) in ethanol (0.005–0.100 mg/mL). The results are expressed as mg_eq-L-alanine_/g_b_.

#### 2.2.4. Determination of Proteins Content

The protein concentration was measured by the reduction reaction of Cu^2+^ to Cu^+^ by proteins in an alkaline medium (Biuret reaction). The reaction was followed by colorimetry detection of Cu^+^ using a reagent that contains bicinchoninic acid (BCA). A volume of 200 μL of kit commercial Pierce BCA Protein Assay reagent was added to 25 μL of supernatant of each biomass. The sample was shaken and incubated at 27 °C for 30 min. Later, the absorbance at 562 nm was measured using distilled water as blank and compared with a standard curve (see Supplementary Materials) made with albumin (≥98%) in ethanol (0.06–2.00 mg/mL). The values are given as mg_eq-albumin_/g_b_.

#### 2.2.5. Determination of Total AST Content

For each biomass type (*bU*, *bPEF*, *bPEF + I*), 0.03 g of dried solid was added to 1 mL of dimethyl sulfoxide (≥99.7%) (DMSO), and the yeast cells were mechanically disrupted using a bead mill (Mini-Beadbeater-Plus; BioSpec, Bartlesville, MO, USA). Each sample was then centrifuged at 6000× *g* for 10 min, and the resulting liquid phase was analyzed by UV−vis spectroscopy. The total AST per gram of dry biomass (*W*_T,AST_) was calculated using the following equation:

W_T,AST_(mg_AST_/g_b_) = (V·A_480_ × 10^6^)/(E^1%^ × 100·M) (1)

where *V* is the solvent volume (mL); *A*_480_ is the absorbance at 480 nm; *M* is the dry biomass mass (g); and *E*^1%^ is the specific absorptivity of AST in DMSO (*E*^1%^ = 2100). The experiments were replicated twice.

#### 2.2.6. Determination of Antioxidant Capacity

The DPPH assay was used to assess the antioxidant capacity of the supernatants from different biomass types. The method is based on monitoring the reduction reaction of the 2,2-diphenyl-1-picrylhydrazyl (DPPH) radical via spectroscopy at 516 nm [25]. A DPPH solution was prepared in methanol at a concentration of 0.04 g/L. Equal volumes of the DPPH solution and each biomass supernatant were mixed and incubated in darkness for 30 min. Subsequently, the absorbance at 516 nm was measured, using a 50% *v/v* methanol-water solution as the blank and a 50% *v/v* methanolic DPPH–water solution as the control. A standard curve (see Supplementary Materials) was prepared with Trolox (≥98%) in methanol at concentrations ranging from 0 to 10 μg/L. Results are expressed as mg Trolox equivalents per gram of biomass (mg_eq-Trolox_/g_biomass_).

### 2.3. Extraction of AST from Dry X. dendrorhous Biomass

#### 2.3.1. Hydrophobic Eutectic Solvents Preparation and Characterization

The characteristics of the pure chemicals and hESs solvents are found in the Supplementary Materials. The last ones were the equimolar mixtures of linalool (L), or l-menthol (M), or eugenol (E), or geraniol (G), or cinnamyl alcohol (C), with oleic acid (Oa). The mixture thymol (T) with oleic acid of mole ratio (1:2) was also studied because the equimolar one was solid at 25 °C. The acronyms used throughout the manuscript are LOa, MOa, EOa, GOa, COa, and TOa2. They were prepared by mixing the pure components in the appropriate mole ratio using a PB210S Sartorius balance with an uncertainty in the mass of u(m) = 1 × 10^−4^ g. Later, stirring with gentle heating (50 °C) were applied until a homogeneous liquid was obtained. The water content was measured with a Crison KF 1S-2B (Crison, Barcelona, Spain) automatic titrator using the Karl Fisher method. The values were lower than 250 ppm for all hESs.

To characterize the solvents, properties such as melting temperature ($T_{m}$), density (ρ), speed of sound (u), refractive index ($n_{D}$), and viscosity (η) were determined at 25 °C and 0.1 MPa. The measurements were performed with several devices well known in the bibliography. Briefly, the temperature of the phase change of each hES was determined with a differential scanning calorimeter (TA Instruments DSC Q2000, New Castle, DE, USA). The calibration was performed with a standard sample of Indium and the heating and cooling rate was of 3 K/min. The ρ and u were determined with an Anton Paar DSA 5000 densimeter (Anton Paar, Graz, Austria) and sound analyzer operating to 3 MHz. The device was calibrated with two reference fluids: air and water MilliQ (resistivity of 18.2 μS‧cm^−1^). The $n_{D}$ was measured with a refractometer Dr. Kernchen Abbemat-HP calibrated with water MilliQ. Finally, the kinematic viscosity, ν, was measured with a Schoot-Geräte AVS-440 automatic measurement unit. The dynamic viscosity, η, was calculated as η=ρ·ν. The Supplementary Materials report a summary of the apparatus type and uncertainties for each property. Each apparatus was checked with benzene (99.8%) and the mean relative deviations (MRD(X)/%) obtained by comparison between our data and those from the literature were MRD(ρ)*=* 0.004%, MRD(u) = 0.026%; MRD($n_{D}$) = 0.007%; MRD(η) = 0.28%.

#### 2.3.2. Extraction of AST from Biomass

To measure the AST mass extracted with hESs as the solvent, 0.02 g of the biomass and 5 mL of the solvent were incubated with shaking at constant temperature (*T*) for a fixed time (*t*). The *T* and *t* values were different depending on the experiment conditions. After centrifugation at 6000× *g* for 20 min, aliquots of solvent containing the extract were taken and analyzed. A concentration process was not necessary. Each extracted sample was analyzed after diluting it in ethanol to compare it with the standard curve and each extraction was performed twice. The AST content in terms of extracted mass of AST per gram of dry biomass, *W*_AST_(mg_AST_/g_b_), was quantified by UV−vis after centrifugation (2390× *g*, 10 min). The analysis procedure was validated by us in a previous paper [16] for this specific biomass. Spectra were obtained with a VWR 6300 PC (VWR, Radnor, PE, USA) double-beam equipment (u(λ) = ±0.2 nm) carrying out a scanning from 400 to 600 nm. The absorbance in the maximum peak was taken and compared with a calibration curve of standard tAST in ethanol (1.5–10 ppm). Each analysis was duplicated and each sample was diluted in ethanol twice. Residual biomass was not analyzed.

### 2.4. Data Treatment

#### 2.4.1. Experimental Design and Mathematical Modeling

Once the optimal solvent was chosen, a Central Composite Design (CCD) was used to evaluate the influence of the l-menthol mole fraction (*x*_M_ = 0.4–0.6), temperature (25–35 °C), and extraction time (4–24 h) on the release of AST from the three dry biomasses assayed. The effect of the parameters was evaluated by the response surface methodology (RSM) fitting experimental data to the following equation:

(2) $$Y=\beta_{0}+\sum_{i=0}^{3}\;\beta_{i}\;X_{i}+\sum_{i=0}^{3}\;\sum_{j=0}^{3}\;\beta_{ij}\;X_{i}\;X_{j}$$

where *Y* is the response; *X_i_* and *X_j_* are the independent variables; *β*_0_ and *β_i_* are the intercept and the linear regression coefficients, respectively; and *β_ij_* indicates the crossing (*i* ≠ *j*) and the quadratic (*i* = *j*) coefficients.

#### 2.4.2. Statistical Analysis

The present results are expressed as the mean ± standard deviation of four replicates. The means were compared by the Tukey test at a 5% significance level using Microsoft Office Excel 2016 software. The CCD, multiple regression analysis, and model significant evaluations were performed with Design Expert Ver. 23.1.1 software (Stat-Ease Inc., Minneapolis, MN, USA). The one-way analysis of variance (ANOVA) was used to statistically analyzed the significance of the regression model performing the F test with a 95% (α = 0.05) level of significance. Various statistical parameters indicate the adequacy of the model: *p*-values of each coefficient have to be lower than 0.0001, *F*-value higher than 12, adequate precision higher than 4, coefficient of variation (*C.V.*) lower than 10%, and the difference between the adjacent and predicted regression coefficients ((*R*^2^)_adj_–(*R*^2^)_pred_) lower than 0.2. In the optimization process, a value of the desirability function near to 1 is preferred.

### 2.5. Molecular Dynamics

Molecular dynamics simulations were performed using Assisted Model Building and Energy Refinement software (AMBER18) and General Amber Force Field (GAFF) [26]. The initial cubic simulation boxes were obtained using the Packmol program [27], and pdb archives of each compound were generated using Maestro software (v. 2018-4, New York, NY, USA). Long-range interactions were treated using the Ewald method. Van der Waals interactions were described by a Lennard-Jones potential and a cutoff distance of 10 Å was applied. Periodic boundary conditions (PBC) and the leap-frog algorithm were employed for the simulations. All simulations were carried out following several steps. First, the energy of the system was minimized to avoid spatial constrains of high energy that can disrupt the simulation. Then, the system was gradually heated from −273 °C to 27 °C. Temperature was controlled by Langevin dynamics [28]. This was followed by a density equilibration and a final equilibration step that includes the monitoring of several structural properties. The last two steps were carried out under NPT conditions. Once the system was equilibrated, two consecutive productions (10 ns) were performed. Then, the analysis of the trajectories was performed using the VMD [29] and CPPTRAJ programs [30].

## 3. Results and Discussion

### 3.1. Characterization

#### 3.1.1. Biomass Characterization

The different types of biomasses (*bU*, *bPEF*, *bPEF + I*) have been characterized by determining the content of solids, GSH, FAN, proteins, total AST content, and the antioxidant capacity. A sample of yeast supernatant whose cells have been mechanically destroyed (*bead-mill*) has also been included in this study. The numerical results are found in the Table 1.

The highest value of dry weight was obtained in the *bU* biomass. The treatment of biomass with PEF resulted in greater release of water-soluble compounds compared to the control owing to the membrane permeabilization. This effect was intensified by incubating the treated biomass for 24 h (*bPEF + I)* since the compounds had more time to release. As expected, the lowest value was found for the *bead-mill* sample because this treatment type result in the total cell rupture. Regarding glutathione content, no GSH was detected in the *bU* and *bPEF* biomasses. In *bPEF + I*, the amount obtained was much lower, less than 14%, than that of the bead mill sample. According this, the electroporation would be an ineffective treatment for obtaining GSH from yeast but the incubation added to the PEF could be used in the revaluation of biomass. The FAN content was higher in the biomasses subjected to treatment than in the *bU*. Nevertheless, the mechanically treated biomass presented a lower value than those subjected to PEF. Specifically, the FAN content in *bPEF + I* biomass was 2.7 times that of the *bead-mill.* This ratio was found by Jacob et al. [31] in autolyzed yeast suspensions. In the literature, it has been reported that electroporation accelerates autolysis in yeast by increasing its enzymatic activity [32,33]. Thus, the large FAN content in *bPEF + I* would be due to the hydrolysis of proteins in yeast cells catalyzed by proteases during incubation. The protein content in the supernatant of the *bead-mill* biomass was much higher than the others, showing the low efficiency of the PEF treatment in the extraction of these compounds.

It seems that electroporation does not permeabilize cells large enough to release great macromolecules. Despite this, a slight effect of electroporation and incubation was observed. In relation to the protein amount in the *bU* biomass, the content in *bPEF* and *bPEF + I* was double and triple, respectively. From the values of the total AST content in the biomass, the extraction efficiency (*EE*_AST_ = 100·*W*_AST/_*W*_T,AST_)can be obtained, and a comparison with different methods could be discussed. An opposite sequence to that of the dry extract was observed in the carotenoid concentration of the biomass. As a result of electroporation, the dry extract of *bPEF* and *bPEF + I* biomasses was lower due to the leakage of cytoplasmic components. Therefore, the AST mass was higher in both treated samples, especially in the one also subjected to incubation, than in the control. The effect was more pronounced in the PEF-treated and incubated biomass. The antioxidant capacity of the biomasses is provided by several compounds such as AST, GSH, amino acids, and proteins. The greater the soluble compounds content in the supernatant, the greater its antioxidant activity. Therefore, it is logical that this parameter follows a sequence opposite to that obtained for dry weight.

#### 3.1.2. Hydrophobic Eutectic Solvents Characterization

Knowing the values of the thermodynamic and transport properties of the solvent used in an extraction process is basic for its operational design. The standard melting point (*T*_m_) indicates the lowest temperature at which the solvent remains in the liquid phase at 0.1 MPa. The *ρ* and *η* data are necessary for the calculation of engineering parameters such as the Reynolds number. Properties derived from the previous ones allow to analyze the process based on the compaction of the fluid and its affinity for the solute.

Table 2 shows the values of the experimental *T*_m_ and that assuming ideal behavior (*T*_m_^id^). The latter was calculated from the Schraeder equation [34] taking the activity coefficient as unity. The smaller the difference between both values, the more ideal the system is. Our systems showed high ideality; that is, the interactions between all the molecules in each of our mixtures were similar. Moreover, the LOa mixture presented the deepest liquid window and GOa and TOa2 could give rise to solidification problems at room temperature. The values of other thermophysical properties measured at 25 °C and 0.1 MPa are also collected in Table 2.

All hESs were less dense than water with a difference greater than 50 kg·m^−3^. Therefore, all could be suitable solvents in two-phase processes. EOa was the denser liquid and sound traveled faster in COa. Both properties allow estimating fluid compaction through isentropic compressibility (*κ*_S_) and free intermolecular length (*L_f_*). The calculated values indicated that the most and least compact fluids were COa and LOa, respectively. The *n*_D_ values were also agreed with these results. From *n*_D_ and *ρ*, the molar refraction (*R*_m_) was calculated. This property indicates the polarizability of a mole of fluid and therefore its affinity with a solute. Also, it is related to the volume occupied by a mole of molecules. Then, the free volume (*f*_m_) can be calculated as the difference between *R*_m_ and the molar volume. The *f*_m_ values are those expected, taking into account that, for similar chain lengths, cyclic and branched compounds such as C, T, E, M, and L are more voluminous than linear ones and that the structure of the aromatic ring included in C, T, and E is flat. The *η* is another essential parameter in the characterization of a solvent. The higher the viscosity, the more difficult it is for molecules to move between fluid layers. This means that mass exchange can be impaired. One way to overcome this problem is to force movement through external agitation. In our case, the solvents were not very viscous. Although one of the components of our hESs was common, the viscosity of the mixtures was very different. They ranged between 16.19 mPa·s of GOa and 30.10 mPa·s of MOa. The non-planar structure of the menthol cyclic ring and the branching of its substituent groups means that the MOa mixture presents greater steric hindrance than the other hESs, which have components that are linear or aromatic ring-containing.

### 3.2. Extraction of AST from X. dendrorhous Freeze-Dried Biomass Using Different hESs

**Figure 2:**
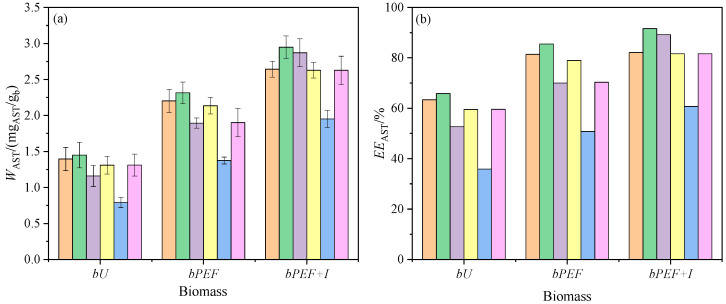
Extracted mass of AST per gram of dry biomass, *W*_AST_/(mg/g_b_), from freeze-dried untreated (*bU*), PEF treated (*bPEF*) and PEF treated and subsequent incubation (*bPEF + I*) biomass of *X. dendrorhous* with several hydrophobic eutectic solvents (hESs) at a temperature of 25 °C and an extraction time of 6 h. (**a**) Extracted mass; and (**b**) extraction efficiency. (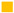), LOa; (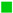), MOa; (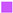), EOa; (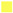), GOa; (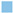), COa; (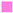), TOa2. Error bars, 95% C.I.

In this section, a preliminary screening of hESs to select the best oleic-based eutectic solvent in the AST recovery from yeast is presented. The mixtures of hESs and biomass were stirred for 6 h into an incubator at 25 °C. The *W*_AST_ and *EE*_AST_ values of the extraction experiments are shown in Figure 2a and 2b, respectively (see Supplementary Materials to find the numerical values). In solid–liquid extractions, several consecutive steps take place. First, the solvent has to move from the bulk of the solution to the surface of the solid. After, the solvent has to penetrate into the pores and dissolve the solute. Finally, the extracted solute has to migrate to the bulk of the liquid. Any of these steps may be the one that limits the efficiency of the process. Therefore, both the preparation of the solid matrix and the choice of the suitable solvent and conditions are parameters to evaluate to improve the extraction results.

#### 3.2.1. Effect of the Pulsed Electric Field (PEF) on the Extraction Process

A pretreatment of the solid matrix can facilitate solute–solvent contact. Reducing the particle size increases the interfacial contact area, although excessive reduction can cause unwanted compaction of the solid. The removal of moisture increases the solute–hydrophobic solvent ratio. Chemical changes using enzymes can be induced in the cells containing the solutes. Additionally, physical changes to the cell wall can be caused by the application of pressure, temperature, or electrical fields [35]. In this section, the influence of the latter on the efficiency extraction with hESs is analyzed. For all hESs, the process was more efficient in the pretreated biomasses. The extracted AST mass in the *bPEF* biomass was up to 1.74 times that of *bU*. Adding the incubation stage, the ratio *W*_AST_(*bPEF + I*)/*W*_AST_(*bU*) ranged from 1.85 to 2.47 (see Supplementary Materials). The positive effect on the AST extraction of pulse application, especially if a subsequent incubation period is included, has already been previously observed regardless of the type of biomass (wet or dry) and the nature of the solvent (hydrophilic or hydrophobic) [16,36]. The results can be explained considering that the membrane electroporation favors the leakage of cytoplasmic compounds through the lipid bilayer (Section 3.1.1). This fact would cause a decrease in intracellular osmotic pressure with the later release of AST to the cytoplasm by plasmolysis of the lysosomes. The hydrophobic nature of the hESs would allow them to cross the membrane and interact with AST efficiently.

#### 3.2.2. Effect of the hESs Composition on the Extraction Process

The extraction profile was the same for the three biomasses type (Figure 2). The mixture with C was the least effective solvent. For COa, the *EE*_AST_ value in the *bPEF + I* biomass was lower than that obtained for most of the mixtures in the *bU*. On the other hand, MOa was the most effective solvent with *EE*_AST_(*bU*) = 62.2%, *EE*_AST_(*bPEF*) = 83.6%, and *EE*_AST_(*bPEF + I*) = 89.4%. These efficiency data were higher than those previously published using ethanol or thymol-based eutectics as solvents [16]. This fact was expected due to the greater hydrophobicity provided by oleic acid. The complete sequence of efficiency of solvents was COa < EOa < TOa2 ≈ GOa < LOa < MOa.

The ability of a solvent molecule to interact with a solute molecule within a matrix depends on both its chemical affinity and the possibility of their encounter. Considering the structure of AST, the first factor will be greater the larger the hydrophobic region of the solvent. For the second, solvents with much free volume and low surface tension favor the accommodation of the large molecules. Additionally, a low-viscosity solvent facilitates movement between layers. In relation to the affinity, the hESs used contain a common component so it can be assumed that the observed differences will be given by the other component. The partition coefficients of the components of our mixtures are published in the literature (see Supplementary Materials). According to these values and the near-ideal behavior observed in Section 3.1.2, the hydrophobicity sequence would be COa <EOa < LOa < MOa ≈ TOa2 < GOa. Regarding the ease of the solvent to form cavities, the compaction sequence (Table 2) was COa < EOa < TOa2 < GOa < LOa ≈ MOa.

According to the above, the least hydrophobic solvents were the least extractive, and among the most hydrophobic, those that were least compact were the most effective. It should be noted that MOa was also the most viscous solvent, and therefore, the one that exhibits the greatest difficulty in transferring matter within the liquid. However, the continuous stirring minimized this fact. It seems that the free volume into the bulk of the fluid was the determining factor in the extraction process.

To compare our results with those obtained in other studies, several factors must be taken into account: the source of AST, the type of pretreatment carried out on the biomass, the nature of the solvent, and the parameters of the extraction procedure. We obtained [16] a maximum *EE*_AST_ = 79% after stirring for 24 h at 30 °C a mixture of dry yeast pretreated (PEF and incubated) and thymol:salol ($x_{T}$ = 0.3) ES. Mussagy et al. [13] achieved an *EE*_AST_ = 81.8% after stirring for 1 h at 65 °C from wet yeast (no pretreated) with the choline chloride:butyric acid (1:5, molar ratio) eutectic solvent. This group has also studied the AST extraction with ionic liquids from *H. Pluvialis* [37]. From this seaweed and ESs similar to ours, Pitacco et al. [38] obtained an *EE*_AST_ = 60% after 6 h of extraction and at 60 °C. All above values are lower than those found by us in this work. Higher efficiencies (87.4% to 124.16%) were achieved from microalgae using CO_2_ in both the supercritical state and expanded with ethanol [39,40]. Also, the application of ultrasound in a biphasic solvent extraction system (2-propanol:ammonium sulfate) increased the extraction efficiency of AST by up to 99% [41]. However, the cost of the necessary equipment and the use of the problematic solvents are a great drawback in the implementation of both techniques in the industry.

### 3.3. Evaluation of the Extraction Conditions of AST from X. dendrorhous Freeze-Dried Biomass Using the Best Solvent

Once the MOa mixture was chosen as the best solvent, an optimization of the extraction conditions was carried out using an RSM approach. A 17-run CCD experiment was performed with three variables. The factors’ values were chosen to avoid problems of both solidification of the mixtures and decomposition of the AST. Thus, the compositions as menthol mole fraction in MOa (*x*_M_) were 0.4, 0.5, and 0.6. The extraction temperatures (*T*) studied were 25 °C, 30 °C, and 35 °C. This *T* range was chosen around room temperature so that it was not necessary to cool the system, a process that is generally more expensive than gentle heating. The extraction times (*t*) in the experiments were 4 h, 14 h, and 24 h. The characteristics of each trial and the results obtained are collected in the Supplementary Materials.

As in the previous section, the biomass pretreatment caused a considerable improvement in the extraction process, which confirms the effectiveness of both the PEF treatment and the incubation stage. The extracted *W*_AST_ from different biomasses were between 0.967 and 1.908 mg_AST_/g_b_ for *bU*, 1.847 and 2.411 mg_AST_/g_b_ for *bPEF*, and 2.333 and 2.970 mg_AST_/g_b_ for *bPEF + I*. For similar extraction conditions, the *W*_AST_(*bPEF*)–*W*_AST_(*bU*) ranged from 0.42 to 1.12 mg_AST_/g_b_. The improvement in *bPEF + I* biomass relative to *bU* was up to 1.92 mg_AST_/g_b_. This highlights that the *W*_AST_(*bPEF + I*) at *t* = 4 h was similar to *W*_AST_(*bPEF*) at *t* = 24 h and much greater, up to 200%, than *W*_AST_(*bU*). The longer the extraction time, the greater the mass extracted. The improvement of *W*_AST_ with increasing *t* was greater in untreated biomass. In terms of efficiency, the effect of pretreatment was minimized with longer *t* obtaining in all cases high *EE*_AST_ values at 24 h. They ranged from 74% (*x*_M_ = 0.4, *T* = 35 °C) to 92% (*x*_M_ = 0.5, *T* = 30 °C). In relation to the effect of *T*, a maximum near to 30 °C was observed for all biomasses. This fact was more marked in that with a highest total amount of AST (*bPEF + I*). Generally, an increase in temperature facilitates the solubilization. However, in the most sensitive compounds, there may be risk of thermal decomposition. The literature reports a non-negligible decomposition of the AST in the temperature range of our work [42], although at longer times than those used here. This fact could explain the maximum extraction obtained at an intermediate temperature. No clear trends of *x*_M_ on *W*_AST_ were found. The results were statistically evaluated and fitted according to Equation (2). The extracted mass of AST per gram of dry biomass, *W*_AST_(mg_AST_/g_b_), can be obtained from the following second-order polynomials:

*W*_AST_(*bU*) = 1.5928 − 1.5517·*x*_M_ + 0.0128·*t* + 0.1136·*x*_M_·*t* − 1.12 × 10^−3^·*t*^2^ (3)

*W*_AST_(*bPEF*) = 1.0363 + 0.0266·*T* + 0.0824·*t* − 1.47 × 10^−3^·*T*·*t* − 7.05 × 10^−4^·*t*^2^ (4)

*W*_AST_(*bPEF + I*) = −6.1684 + 0.5362·*T* + 0.1250·*t* − 3.87 × 10^−3^·*T*·*t* − 7.96 × 10^−3^·*T^2^* (5)

The statistical analysis of variance (ANOVA) conducted for each biomass type is reported in the Supplementary Materials. The model was significant in all cases with F-values much higher than the minimum required value (Section 2.4.2). In addition, the lack of fit was not significant in relation to the pure error. The higher the F-value of the variable, the greater its weight in the model. Then, *t* was the most influential factor in *bU* and *bPEF* biomasses, and the crossing factor *T*·*t* was in *bPEF + I*. On the other hand, *T* was non-significant on the AST extraction from *bU* biomass and *x*_M_ was not in all cases.

**Figure 3:**
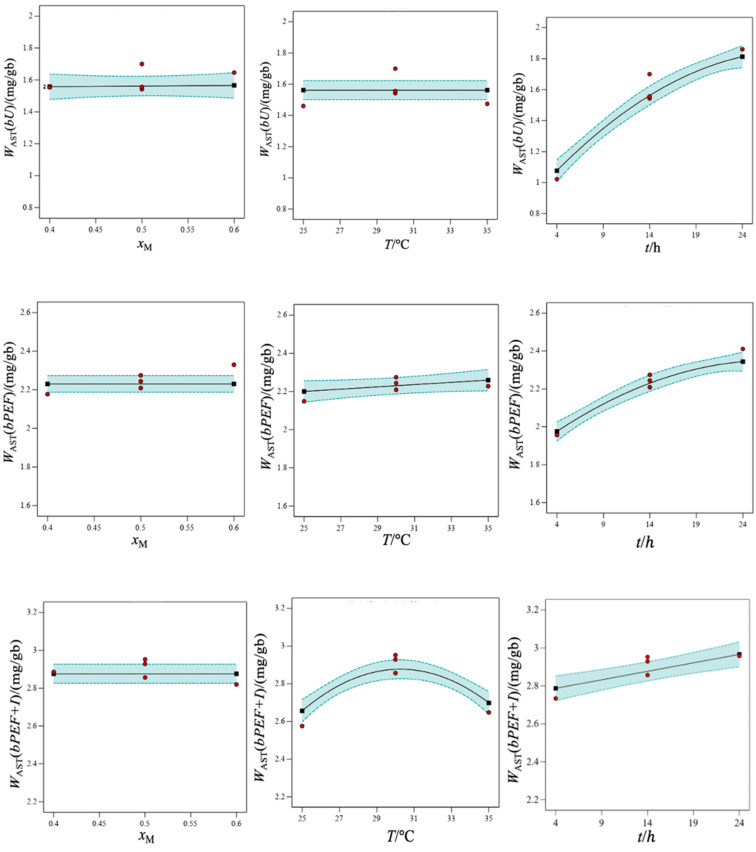
Extracted mass of AST per gram of biomass, *W*_AST_/(mg/g_b_), from freeze-dried untreated (*bU*), PEF treated (*bPEF*), and PEF treated and subsequent incubation (*bPEF + I*) biomass of *X. dendrorhous* using mixtures of l-menthol (M) and oleic acid (Oa) as the solvent. Effect of different factors on the extraction efficiency: l-menthol mole fraction (*x*_M_) at *T* = 30 °C and *t* = 14 h; temperature (*T*) at *x*_M_ = 0.5 and *t* = 14 h; extraction time (*t*) at *x*_M_ = 0.5 and *T* = 30 °C.

Figure 3 shows the effect of the individual variables on the extracted mass taken; intermediate values of the rest of the factors are displayed. The *W*_AST_–*x*_M_ slope was practically zero, which shows the small effect of the hES composition. The variation of *T* caused a higher change on the extraction efficiency in the pretreated biomasses, especially in *bPEF + I* in which a maximum marking was observed. Finally, the highest *W*_AST_–*t* slope was found in the experiments with *bU* and the lowest in *bPEF + I* biomass. A numerical diagnosis of the adequacy of the model can be carried out with the values of the signal-to-noise ratio (adequate precision), regression coefficients of the equations, and the coefficient of variation (see Supplementary Materials). All of them were within the recommended values (Section 2.4.2) showing reproducibility and accuracy of the experimental data. A graphical diagnosis of the model with the predicted versus actual data for each biomass is found in the Supplementary Materials. The mutual interactions between parameters can be analyzed from 3D surface plots and 2D contours. Figure S3a displays the joint effect of the composition and extraction time of *bU* at *T* = 30 °C. The cross-interaction was greater at higher values of both factors as shown by the greater curvature of the contour in this region. In addition, Figure S3b,c report the cross-effect of *T* and *t* from the pretreated samples at *x*_M_ = 0.5. The interaction in both biomasses was minimal at the lowest *t* and *T*. It was maximum at the highest *t* and lowest *T* for *bPEF* and at the highest *t* and intermediate *T* for *bPEF + I*.

Determining the best extraction conditions, minimizing some of the determining factors of the process, is a calculation of interest in the industry. Previously, the RSM approach indicated that composition was a non-significant factor in our process so the optimization will focus on *T* and *t*. For the lowest temperature studied, 25 °C, the minimum time to obtain an adequate extraction efficiency was close to 13 h for all biomasses. Then, the values predicted were *EE*_AST_(*bU*) = 69.0%, *EE*_AST_(*bPEF*) = 80.1%, and *EE*_AST_(*bPEF + I*) = 82.4%. For the lowest time, 4 h, the maximum efficiency from untreated biomass was 54% at 35 °C, and from treated biomass was *EE*_AST_(*bPEF*) = 73.1% and *EE*_AST_(*bPEF + I*) = 87.1% at 30 °C. The best conditions for each biomass and their efficiencies were *EE*_AST_(*bU*) = 71.5% at 29.4 °C, 12.7 h, *x*_M_ = 0.496, *EE*_AST_(*bPEF*) = 87.4% at 35.0 °C, 21.3 h, and *x*_M_ = 0.512, and *EE*_AST_(*bPEF + I*) = 87.8% at 26.3 °C, 15.3 h, and *x*_M_ = 0.434.

The separation of the extract from the solvent after the solid–liquid extraction is a process that usually entails extra expenditure in energy consumption and product use, with the consequent increase in waste [8,11,12]. All these issues are contrary to the philosophy of “Green Chemistry”. A promising alternative consists of using solvents whose properties improve those of the extract, also allowing the field of application of the final formulation to be expanded. We agree with this working approach so we propose the use of the AST/MOa final formulation. To use it in the food, cosmetic, and pharmaceutical fields, both potential adverse effects on the organisms and functional properties of the solvent must be considered. The literature reports low toxicity of the mixtures of M and Oa. Kongpol et al. [43] analyzed the cytotoxicity of MOa mixture obtaining a cell viability higher than 80%. Valente et al. [44] determined a median effective concentration of EC_50_ = 0.83 for this solvent. The MOa mixture exhibited an anti-inflammatory effect increased when it was enriched with curcuminoids. In addition, cytotoxicity of the latter decreased in the presence of MOa [43]. Mussagy et al. [21,45] extracted AST from yeast with bio-based and eutectic solvents. The resulting carotenes-enriched mixtures were used for the production of soap and biofilms with high antioxidant and antimicrobial power.

### 3.4. Analysis of the Interaction of AST–MOa by Molecular Dynamics (MD)

The efficiency of extraction can be conditioned by different factors such as the physicochemical properties of the solvent and the specific solute–solvent interactions. Despite the effectiveness shown by ESs as solvents of active principles, the dissolution mechanism has not yet been elucidated. The most accepted theory is that the groups responsible for H-bond formation in the ESs facilitate interactions with the drug, thereby enhancing the stability of the solute–solvent system. In this section, an assessment of the main interactions established between the compounds of the MOa eutectic mixture and AST is presented. The CPPTRAJ program was used to perform a cluster analysis in which the non-covalent interactions (NCIs) can be visualized as surfaces within the sample. The clustering command provided the disposition of the corresponding aggregate maintained during the maximum number of frames. For this, the electron density (ρ) and the reduced density gradient (RDG or s) are calculated and pictured. Weak interactions like van der Waals provide low ρ and strong interactions like hydrogen bonds or steric hindrance are related to high ρ. Introducing the sign of the second Hessian eigenvalue of the density ($\lambda_{2}$) allows us to differentiate the latter. Attractive interactions have ρ > 0 and $\lambda_{2}\;$< 0, weak interactions have ρ≈ 0 and $\lambda_{2}\approx \;$0, and repulsive interactions have ρ > 0 and $\lambda_{2}\;$> 0. Then, the representation of RDG versus the $\left(\lambda_{2}\right)\rho$ product is a picture that shows the qualitative extent of the interactions through a color code.

**Figure 4:**
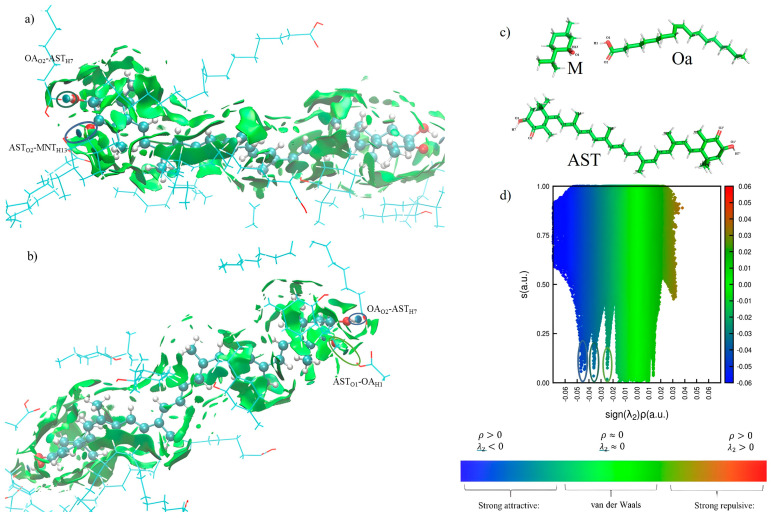
Visualization of reduced density gradient (RDG) of astaxanthin (AST) in a l-menthol (M) and oleic acid (Oa) system. (**a**,**b**) 3D-representation of each of the polar heads of AST; (**c**) atom labeling; (**d**) 2D-representation.

In our system, we identified 10 different AST/MOa clusters and studied the most stable one obtaining the size and the NCI index [46]. The size of the most stable cluster was 0.151 (ranging from 0 to 1); that is, the studied aggregate presented that disposition for 15.1% of the simulation time. The NCI index allowed evaluating the most representative interactions between the MOa mixture and AST. To clarify, the analysis of NCIs between AST and MOa is presented in separate figures (Figure 4a–c), each one showing one of the polar heads of the AST molecule. Each hydrogen bond (H-bond) is represented as an ellipse to improve its visualization and van der Waals interactions as green surfaces.

Briefly, the establishment of three types of H-bonds and different extensive surfaces of hydrophobic van der Waals interactions were observed. Closer examination showed that an H-bond was formed between the hydroxyl hydrogen of M and the carbonyl group of AST (AST_O2_-M_H13_). In addition, van der Waals interactions were observed between aliphatic hydrogens of M, mainly from methyl and isopropyl groups, and aliphatic and olefinic hydrogens of AST. When both molecules were aligned perpendicular, interactions between M hydrogens and methyl groups and olefinic hydrogens of AST were detected. In parallel positioning, the hydrogens of M interacted both with the hydrogens of AST and with the π system of its double bonds through CH-π interactions. Regarding Oa, two H-bonds (Oa_O2_-AST_H7_, AST_O1_-Oa_H1_) were established because the acid acted as the hydrogen bond acceptor and donor. Furthermore, van der Waals interactions near the polar head of AST were observed. These interactions were also present when Oa established H-bonds with polar groups of AST. Also, Oa interacted in parallel alignment with double bonds of AST via its double bond (π-π interactions). An H-bond between M and Oa was also formed (M_O1_-Oa_H7_) when Oa and AST were interacting via the H-bond. This fact would contribute to increase the stability of the AST–solvent system. Figure 4d shows the 2D representation of the interactions in the most stable cluster. This highlights that, although hydrophobic interactions are often neglected compared to hydrogen bonds, they are as important as them in this system, or even more, due to the large aliphatic chains of AST and Oa. M also played a vital role in the establishment of hydrophobic interactions.

## 4. Conclusions

It is known that combination of techniques can improve the extraction efficiency of compounds such as astaxanthin from natural matrices such as yeast. In this work, we propose a pretreatment of biomass with pulsed electric field technology followed by solid–liquid extraction using hydrophobic eutectic solvents. The last ones were binary mixtures of components of essential oils and oleic acid. The electroporation facilitated the release of amino acids and astaxanthin, although it was not sufficient to allow the extraction of larger molecules such as glutathione and proteins. Furthermore, the solvents studied were effective extractants reaching, in most cases, values higher than 80% for the pretreated biomasses. The maximum efficiency was obtained from pretreated and incubated biomass using the l-menthol:oleic acid system as the solvent. Both the values of the thermophysical properties and the simulations with molecular dynamics showed the importance of the hydrophobic interactions between astaxanthin and the solvent components.

## Figures and Tables

**Table 1:** Biomass characterization ^*a*^

Parameter	Biomass			
	*bU*	*bPEF*	*bPEF + I*	*Bead-Mill*
Dry weight/ (g_dry_/g_wet_)	0.0272 ± 0.0004	0.0248 ± 0.0006	0.0215 ± 0.0006	0.0125 ± 0.0008
Total AST/ (g_dry_/g_wet_)	2.201 ± 0.012	2.704 ± 0.025	3.217 ± 0.020	----
Glutathione/ (mg_GSH_/g_b_)	-----	----	0.882 ± 0.009	6.373 ± 0.110
α-amino acid/ (mg_eq.L-Alanine_/g_b_)	7.34 ± 0.16	37.47 ± 2.82	90.62 ± 5.85	33.65 ± 4.48
Proteins/ (mg_eq.Albumin_/g_b_)	7.48 ± 0.12	16.45 ± 0.74	20.98 ± 5.17	191.50 ± 6.22
DPPH/ (mg_eq.Trolox_/g_b_)	0.332 ± 0.005	0.527 ± 0.017	0.663 ± 0.003	1.065 ± 0.032

^*a*^ Mean ±95% confidence interval.

**Table 2:** Thermophysical properties ^*a*^ of hydrophobic eutectic solvents (hESs). Melting temperature assuming ideal behavior (Tmid)b) and experimental melting temperature (*T_m_*), density (ρ), speed of sound (u), isentropic compressibility (κS) c, free intermolecular length  (Lf) d, refractive index ($n_{D}$), molar refraction (Rm)e, free volume (fm)f, and dynamic viscosity (η) at *T* = 25 °C and *p* = 0.1 MPa.

	hESs					
Property	LOa	MOa	EOa	GOa	COa	TOa2
$T_{m}^{id}/$°C	−2.7	3.67	1.85	---*^g^*	3.85	12.6
*T_m_*/°C	−5.1	−0.23	3.52	1.48	5.51	4.48
*ρ*/kg‧m^−3^	885.12	891.11	944.50	883.92	933.61	908.64
*u*/m‧s^−1^	1387.34	1404.22	1427.46	1418.85	1463.58	1421.59
*κ_s_*/TPa^−1^	587.11	569.10	519.95	561.97	500.04	544.63
*L_f_*/Å	0.479	0.471	0.451	0.469	0.442	0.461
*n_D_*	1.45947	1.45967	1.48421	1.46498	1.49421	1.47411
*R_m_*/cm^3^‧mol^−1^	67.50	67.38	67.67	68.29	64.90	73.74
*f_m_*/%	72.64	72.63	71.38	72.48	70.79	69.23
*η*/mPa‧s	16.87	30.10	18.63	16.19	26.76	25.77

*^a^* Standard uncertainties are: $u\left(T\right)$ = 0.005 °C for ρ and u, and 0.01 °C for the rest of properties. The combined expanded uncertainties (0.95 level of confidence, k = 2) are $U_{c}$(*T_m_*) = 0.05 °C; $U_{c}\left(\rho \right)\;$= 0.05 kg·m^−3^; $U_{c}\left(u\right)$= 0.5 m·s^−1^; $U_{c}\left(n_{D}\right)$= 2 × 10^−5^; $U_{c}\left(\eta \right)$= 1%; $U_{c}(\kappa_{s})$= 0.22 TPa^−1^; $U_{c}(L_{f})$= 0.005 Å; $U_{c}(R_{m})$= 0.004 cm^3^·mol^−1^; $U_{c}\left(f_{m}\right)$= 0.03 cm^3^·mol^−1^. ^*b*^ Calculated from the Schraeder equation [34]. κSc=1/ρu2. Lfd = (91.368 + 0.3565 T(K))10^−8^$\sqrt{\kappa_{S}}$. Rme=nD2−1nD2+2Vm; $V_{m}=M/\rho$. fmf=Vm−Rm/Vm. ^*g*^ No melting data of pure G were found.

## Data Availability

The authors confirm that the data supporting the findings of this study are available within the article and its Supplementary Materials.

## Supplementary Materials

The following supporting information can be downloaded at: https://www.mdpi.com/article/10.3390/foods14132371/s1, Table S1. Pure compounds. Name, acronyms, source, purity, structure, and partition coefficient (log*P*); Table S2. Description of hydrophobic eutectic solvents (hESs): Acronym, composition, molar mass calculated ${\left(M\right)}^{a}$, and appearance at *T* = 25 °C and *p* = 0.1 MPa; Table S3. Summary of the devices used in the thermophysical characterization; Table S4. ANOVA study; Table S5. Extraction efficiency of astaxanthin (AST) from freeze-dried untreated (*bU*), PEF treated (*bPEF*) and PEF treated and subsequent incubation (*bPEF + I*) biomass of *X. dendrorhous* with several hydrophobic eutectic solvents (hESs) at temperature of 25 °C and extraction time of 6 h. Results are presented in terms of extracted mass of astaxanthin (AST) per gram of dry biomass (*W*_AST_) ^*a*^ and extraction efficiency (*EE*_AST_) ^*b*^; Table S6. Extraction efficiency of astaxanthin (AST) from freeze-dried untreated (*bU*), PEF treated (*bPEF*) and PEF treated and subsequent incubation (*bPEF + I*) biomass of *X. dendrorhous* using mixtures of l-menthol (M) and oleic acid (Oa) as solvent. Matrix established by central composite design (CCD) and results presented in terms of extracted mass of AST per gram of dry biomass (*W*_AST_) ^*a*^ and percentage (*EE*_AST_) ^*b*^; Table S7. Fit statistics of the regression models for the results of extraction of astaxanthin from freeze-dried untreated (*bU*), PEF treated (*bPEF*) and PEF treated and subsequent incubation (*bPEF + I*) biomass of *X. dendrorhous* using mixtures of l-menthol (M) and oleic acid (Oa) as solvent; Figure S1. Calibration lines of the biomass characterization. (a) Glutathione content; (b) a-amino acid content; (c) Protein content; (d) DPPH antioxidant capacity; Figure S2. Model validation: predicted—actual data for the extraction of astaxanthin from freeze-dried untreated (*bU*), PEF treated (*bPEF*) and PEF treated and subsequent incubation (*bPEF + I*) biomass of *X. dendrorhous* using mixtures of l-menthol and oleic acid as solvent. Figure S3. 2D–contour and 3D–surface plot representing the interaction effect between the two more significant variables with the third one being constant and equal to the central value.
