# Supplementary material for: Eutectic Mixtures Based on Oleic Acid and Pulsed Electric Fields: A Strategy for the Extraction of Astaxanthin from Dry Biomass of Xanthophyllomyces dendrorhous
Source: Foods. 2025 Jul 4;14(13):2371. doi: 10.3390/foods14132371 (PMC12248501; doi:10.3390/foods14132371)
Supplement: Supplementary file 1 [file foods-14-02371-s001.zip › foods-3686737-supplementary.pdf]

Supplementary material

Eutectic mixtures based on oleic acid and pulsed electric fields: a strategy for the extraction of astaxanthin from dry biomass of *Xanthophyllomyces dendrorhous*

Table of contents	*Page*
Table S1. Characteristic of the pure compounds	S1
Table S2. Description of the hydrophobic eutectic solvents (hESs)	S1
Table S3. Summary of the devices used in the thermophysical characterization	S2
Table S4. ANOVA study	S3
Table S5. Results of the extraction efficiency of astaxanthin (AST) with several hESs	S4
Table S6. Matrix established by central composite design (CCD) and results of the extraction efficiency of AST with several mixtures of l-menthol (M) and oleic acid (Oa)	S5
Table S7. Fit statistics of the regression models for the results of extraction of AST with several mixtures of l-menthol (M) and oleic acid (Oa)	S6
Figure S1. Calibration lines of the biomass characterization	S7
Figure S2. Model validation: predicted−actual data for the extraction of AST with several mixtures of l-menthol (M) and oleic acid (Oa)	S7
Figure S3. 2D–contour and 3D–surface plot	S8

Table S1. Pure compounds. Name, acronyms, source, purity, structure, and partition coefficient (log*P*)

Compound (Acronym)	Cas No.	Purity	Structure	logP^a
Oleic acid^b (Oa)	112-80-1	≥90%	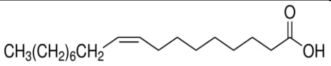	7.64
Linalool^b (L)	78-70-6	≥98.9%	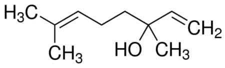	2.97
L-menthol^b (M)	2216-51-5	≥99%	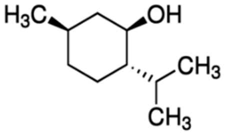	3.20
Eugenol^b (E)	97-53-0	≥99%	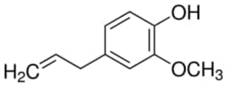	2.49
Geraniol^b (G)	106-24-1	≥98.7%	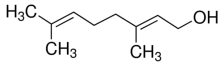	3.56
Cinnamyl alcohol^b (C)	104-54-1	≥98%	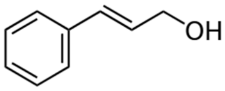	1.70
Thymol^c (T)	89-83-8	>98%	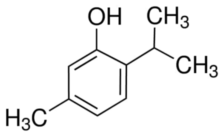	3.30
t-Astaxanthin^b (tAST)	472-61-7	≥99%	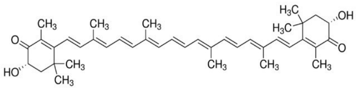	13.27^d

^{a} https://pubchem.ncbi.nlm.nih.gov/ ^{b} Supply by Sigma-Aldrich; ^{c} Thermo Fisher Sci.;
^{d} La J, Kim M, Lee J, Evaluation of solvent effects on the DPPH reactivity for determining the antioxidant activity in oil matrix, Food Science and Biotechnology (2021) 30(3) 367-375.

Table S2. Description of hydrophobic eutectic solvents (hESs): Acronym, composition, molar mass calculated $(M)^a$, and appearance at *T*=25°C and *p*= 0.1 MPa.

hESs acronym	Compound 1 (acronym)	Compound 2 (acronym)	Molar ratio	$M/$ g·mol^{-1}	Appearance
LOa	Linalool (L)	Oleic acid (Oa)	(1:1)	218.36	light yellow liquid
MOa	l-Menthol (M)	Oleic acid (Oa)	(1:1)	219.37	colorless liquid
EOa	Eugenol (E)	Oleic acid (Oa)	(1:1)	223.33	light yellow liquid
GOa	Geraniol (G)	Oleic acid (Oa)	(1:1)	218.36	light yellow liquid
COa	Cinnamyl alcohol (C)	Oleic acid (Oa)	(1:1)	208.31	yellow liquid
TOa2	Thymol (T)	Oleic acid (Oa)	(1:2)	238.38	colorless liquid

$^aM = \sum_i M_i x_i$,

Table S3. Summary of the devices used in the thermophysical characterization.

Property	Devices	$u(T)$/°C	$U_c(Y)^a$	$MRD(Y)^b$/%
T_m	Differential scanning calorimeter, TA Instruments DSC Q2000	0.05	---	---
ρ	Oscillating U-tube density meter, Anton Paar DSA 5000	0.005	0.05 kg·m^{-3}	0.004
u	Sing-around technique in a fixed-path interferometer, Anton Paar DSA 5000	0.005	0.5 m·s^{-1}	0.026
n_D	Standard Abbe refractometer, Abbemat-HP refractometer Dr. Kernchen	0.01	2·10^{-5}	0.007
η	Capillary viscosimeter Ubbelohde, Schoot-Geräte AVS-440	0.01	1%	0.28

$^a k = 2$ (0.95 level of confidence); $^b MRD(Y) = \frac{100}{n}\sum_{i=1}^{n}\left|\frac{Y_{i,lit}-Y_{i,exp}}{Y_{i,exp}}\right|$ checked with benzene

Table S4. ANOVA results of the models for the extraction of astaxanthin from freeze-dried untreated (*bU*), PEF treated (*bPEF*) and PEF treated and subsequent incubation (*bPEF+I*) biomass of *X. dendrorhous* using mixtures of l-menthol and oleic acid as solvent.

Biomass	Variables[a]	Sum of squares	df	Mean square	F-value	*p*-value
bU	Model	1.51	4	0.3784	68.68	< 0.0001
	A-x_M	0.0002	1	0.0002	0.0276	0.8708[b]
	C-t/h	1.35	1	1.35	245.67	< 0.0001
	AC	0.1033	1	0.1033	18.75	0.0010
	C^2	0.0566	1	0.0566	10.27	0.0076
	Residual	0.0661	12	0.0055		
	Lack of Fit	0.0508	10	0.0051	0.6643	0.7318
	Pure Error	0.0153	2	0.0076		
	Cor Total	1.58	16			
bPEF	Model	0.4128	4	0.1032	37.94	< 0.0001
	B-T/°C	0.0088	1	0.0088	3.24	0.0969
	C-t/h	0.3400	1	0.3400	124.99	< 0.0001
	BC	0.0435	1	0.0435	15.99	0.0018
	C^2	0.0205	1	0.0205	7.52	0.0179
	Residual	0.0326	12	0.0027		
	Lack of Fit	0.0305	10	0.0030	2.79	0.2922
	Pure Error	0.0022	2	0.0011		
	Cor Total	0.4455	16			
bPEF+I	Model	0.5467	4	0.1367	36.61	< 0.0001
	B-T/°C	0.0045	1	0.0045	1.21	0.2930[b]
	C-t/h	0.0798	1	0.0798	21.38	0.0006
	BC	0.2992	1	0.2992	80.12	< 0.0001
	B^2	0.1632	1	0.1632	43.72	< 0.0001
	Residual	0.0448	12	0.0037		
	Lack of Fit	0.0399	10	0.0040	1.61	0.4432

[a] l-menthol mole fraction (x_M), temperature (T) and extraction time (t); [b] Term required to support model hierarchy.

Table S5. Extraction efficiency of astaxanthin (AST) from freeze-dried untreated (*bU*), PEF treated (*bPEF*) and PEF treated and subsequent incubation (*bPEF+I*) biomass of *X. dendrorhous* with several hydrophobic eutectic solvents (hESs) at temperature of 25 °C and extraction time of 6 h. Results are presented in terms of extracted mass of astaxanthin (AST) per gram of dry biomass (W_{AST}) [a] and extraction efficiency (EE_{AST}) [b]

hESs	Biomass	W_{AST}(mg_{AST}/g_b)	EE_{AST}(%)
LOa	*bU*	1.396 ± 0.162	63.4
	bPEF	2.201 ± 0.163	81.4
	bPEF+I	2.642 ± 0.111	82.1
MOa	*bU*	1.370 ± 0.177	62.2
	bPEF	2.260 ± 0.152	83.6
	bPEF+I	2.877 ± 0.157	89.4
EOa	*bU*	1.160 ± 0.147	52.7
	bPEF	1.894 ± 0.070	70.0
	bPEF+I	2.517 ± 0.194	78.2
GOa	*bU*	1.310 ± 0.123	59.5
	bPEF	2.135 ± 0.116	78.9
	bPEF+I	2.627 ± 0.108	81.6
COa	*bU*	0.791 ± 0.071	35.9
	bPEF	1.374 ± 0.048	50.8
	bPEF+I	1.952 ± 0.119	60.7
TOa2	*bU*	1.311 ± 0.153	59.6
	bPEF	1.901 ± 0.197	70.3
	bPEF+I	2.627 ± 0.196	81.6
EtOH [c]	*bU*		20.1
	bPEF		37.5
	bPEF+I		57.3

[a] Mean ±95% confidence interval. [b] $EE_{AST}= 100\cdot W_{AST}/W_{T,AST}$; [c] Artigas-Hernández D, Berzosa A, Aguilar-Machado D, Raso J, Artal M, Using eutectic solvents for extracting astaxanthin from dry biomass of Xanthophyllomyces dendrorhous pretreated by pulsed electric fields, Separation and Purification Technology (2023) 324.

Table S6. Extraction efficiency of astaxanthin (AST) from freeze-dried untreated (*bU*), PEF treated (*bPEF*) and PEF treated and subsequent incubation (*bPEF+I*) biomass of *X. dendrorhous* using mixtures of l-menthol (M) and oleic acid (Oa) as solvent. Matrix established by central composite design (CCD) and results presented in terms of extracted mass of AST per gram of dry biomass (W_{AST})^a and percentage (EE_{AST})^b

Run	Variables^c			*bU*		*bPEF*		*bPEF+I*	
	x_M	T (°C)	t (h)	W_{AST} (mg_{AST}/g_b)	EE_{AST} (%)	W_{AST} (mg_{AST}/g_b)	EE_{AST} (%)	W_{AST} (mg_{AST}/g_b)	EE_{AST} (%)
1	0.6	25	24	1.908 ± 0.091	86.8	2.358 ± 0.121	87.4	2.970 ± 0.166	92.2
2	0.5	30	24	1.860 ± 0.205	84.5	2.411 ± 0.162	89.3	2.958 ± 0.173	91.9
3	0.6	35	4	0.967 ± 0.441	44.1	2.089 ± 0.329	77.4	2.886 ± 0.369	89.8
4	0.6	25	4	0.973 ± 0.124	44.1	1.847 ± 0.088	68.5	2.333 ± 0.092	72.4
5	0.5	30	14	1.556 ± 0.299	70.9	2.209 ± 0.278	81.9	2.856 ± 0.290	88.8
6	0.4	25	4	1.167 ± 0.112	53.2	1.911 ± 0.090	70.7	2.466 ± 0.156	76.7
7	0.4	25	24	1.767 ± 0.101	80.5	2.390 ± 0.146	88.5	2.936 ± 0.106	91.3
8	0.4	35	24	1.623 ± 0.144	73.6	2.232± 0.053	82.6	2.611 ± 0.064	81.1
9	0.4	35	4	1.254 ± 0.292	56.4	2.073 ± 0.283	76.7	2.756 ± 0.375	85.7
10	0.5	35	14	1.474 ± 0.142	66.8	2.228 ± 0.075	82.6	2.648 ± 0.109	82.3
11	0.5	30	14	1.542 ± 0.031	70.0	2.275 ± 0.071	84.4	2.928 ± 0.163	91.0
12	0.6	35	24	1.907 ± 0.314	86.8	2.330 ± 0.084	86.3	2.593 ± 0.041	80.4
13	0.4	30	14	1.554 ± 0.217	70.5	2.177 ± 0.233	80.7	2.886 ± 0.224	89.8
14	0.5	25	14	1.460 ± 0.210	66.4	2.149 ± 0.129	79.6	2.575 ± 0.034	79.8
15	0.6	30	14	1.646 ± 0.066	75.0	2.330 ± 0.024	86.3	2.821 ± 0.104	87.6
16	0.5	30	14	1.699 ± 0.067	77.3	2.244 ± 0.184	83.0	2.952± 0.139	91.6
17	0.5	30	4	1.022 ± 0.197	46.4	1.957 ± 0.124	72.6	2.733 ± 0.157	84.8

^{a} Mean ±95% confidence interval. ^{b} $EE_{AST} = 100\frac{W_{AST}}{W_{T,AST}}$; ^{c} l-menthol mole fraction (x_M), temperature (T) and extraction time (t)

Table S7. Fit statistics of the regression models for the results of extraction of astaxanthin from freeze-dried untreated (*bU*), PEF treated (*bPEF*) and PEF treated and subsequent incubation (*bPEF+I*) biomass of *X. dendrorhous* using mixtures of l-menthol (M) and oleic acid (Oa) as solvent.

	bU	*bPEF*	*bPEF+I*
Std. Dev.	0.0742	0.0522	0.0611
Mean	1.49	2.19	2.76
C.V./%	4.97	2.38	2.21
R^2	0.9581	0.9267	0.9243
$(R^2)_{adj}$	0.9442	0.9023	0.8990
$(R^2)_{pred}$	0.9231	0.8623	0.8339
Adeq. Precision	23.9242	18.2523	17.8768

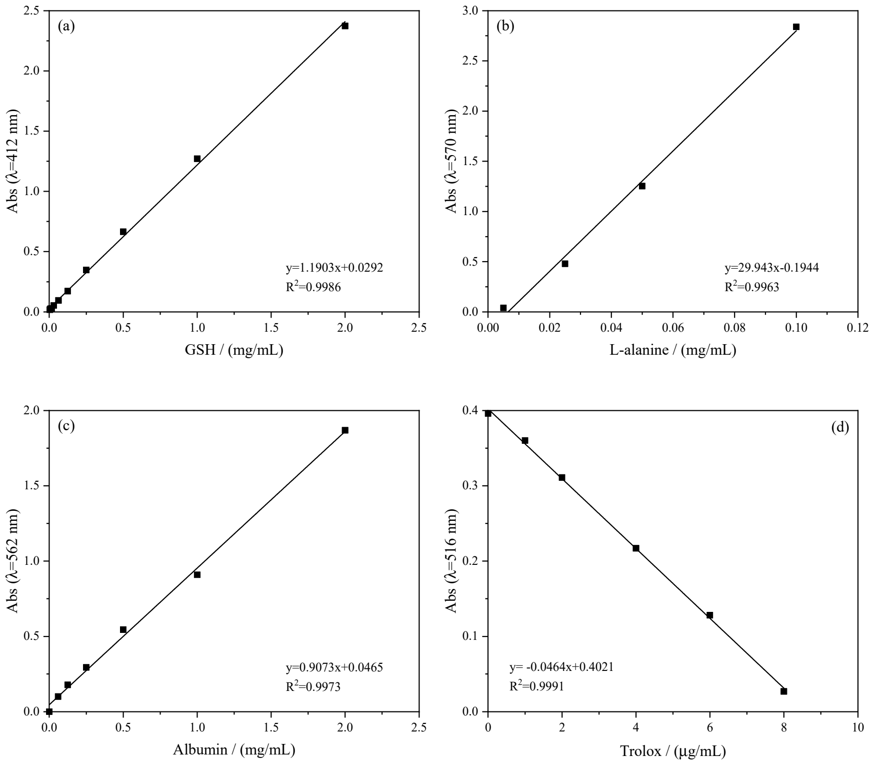

Figure S1. Calibration lines of the biomass characterization. (a) Glutathione content; (b) α-amino acid content; (c) Protein content; (d) DPPH antioxidant capacity

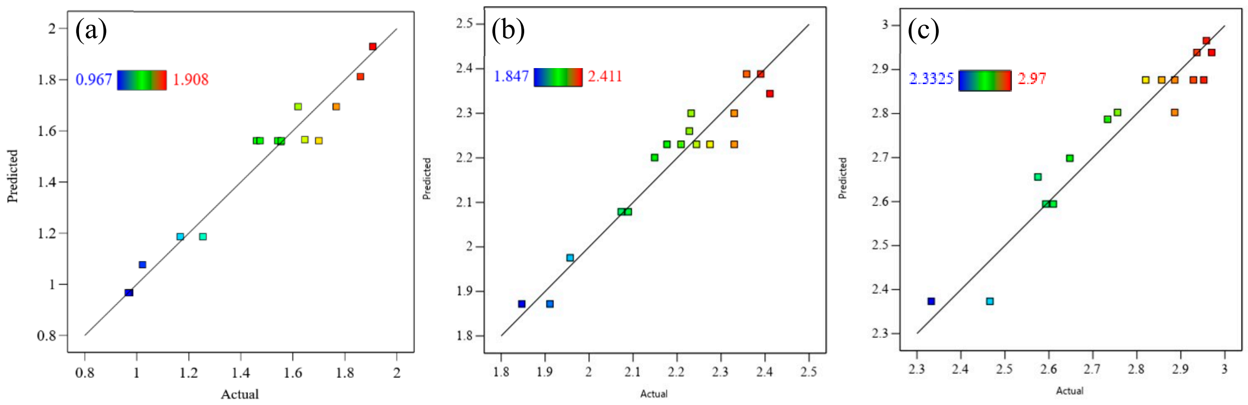

Figure S2. Model validation: predicted−actual data for the extraction of astaxanthin from freeze-dried untreated (*bU*), PEF treated (*bPEF*) and PEF treated and subsequent incubation (*bPEF+I*) biomass of *X. dendrorhous* using mixtures of l-menthol and oleic acid as solvent.

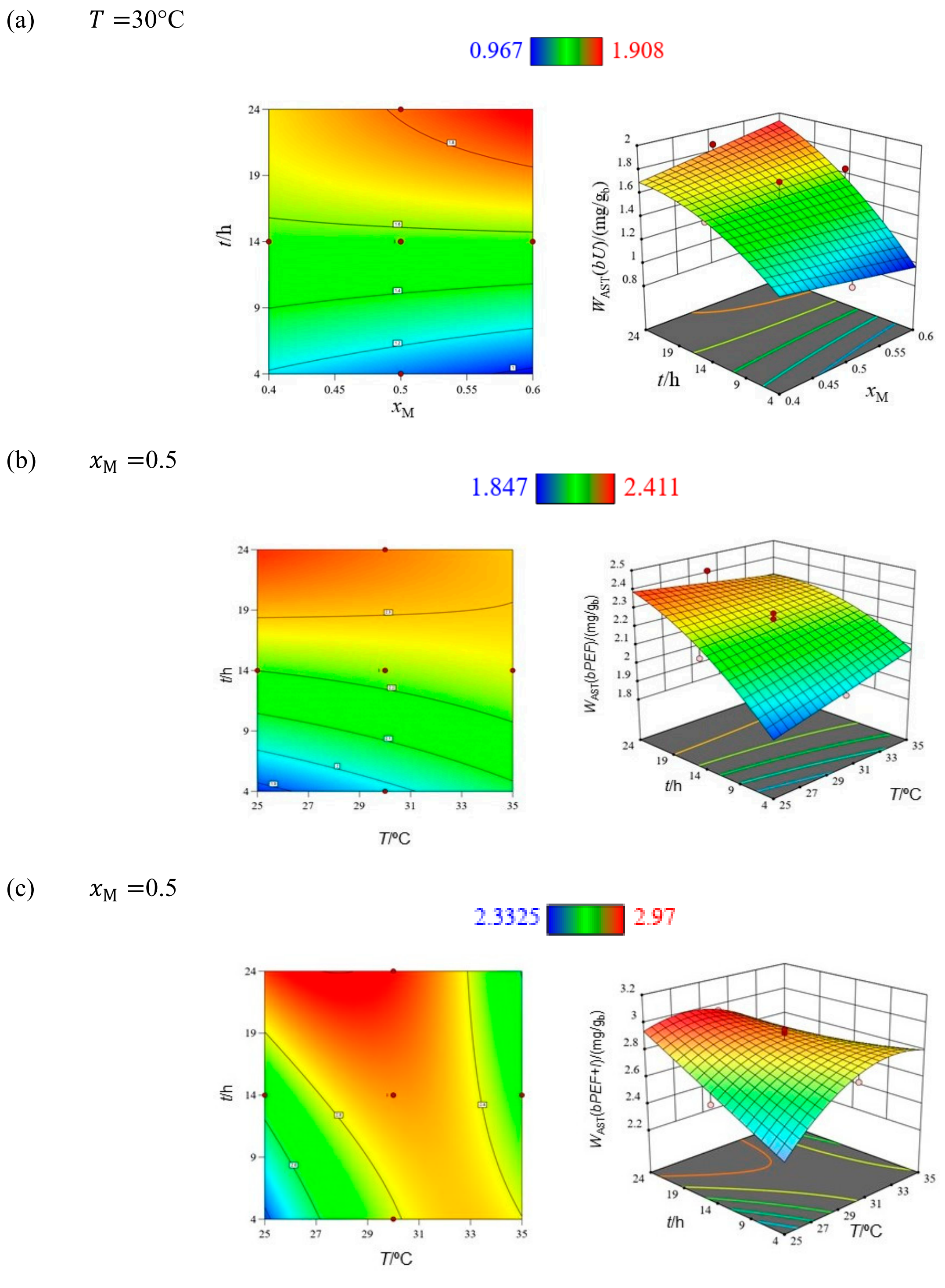

Figure S3. Response 2D–contour and 3D–surface plots representing the interaction effect between the two more significant variables with the third one being constant and equal to the central value. The variables are l-menthol mole fraction (x_M), temperature (T) and extraction time (t). Extracted mass of AST per gram of dry biomass, W_{AST}/(mg/g$_b$), from the three types of biomasses of *X. dendrorhous* using mixtures of l-menthol (M) and oleic acid (Oa) as solvent. (a), freeze-dried untreated (*bU*); (b), PEF treated (*bPEF*); and (c), PEF treated and subsequent incubation (*bPEF+I*).
